# Mortalidade por hanseníase em contextos de alta endemicidade: análise espaço-temporal integrada no Brasil

**DOI:** 10.26633/RPSP.2019.87

**Published:** 2019-11-06

**Authors:** Anderson Fuentes Ferreira, Eliana Amorim de Souza, Mauricélia da Silveira Lima, Gabriela Soledad Márdero García, Francesco Corona, Elaine Silva Nascimento Andrade, Sebastião Alves de Sena Neto, Carmelita Ribeiro Filha, Adriana da Silva dos Reis, Léia Gadelha Teixeira, Alberto Novaes Ramos Jr.

**Affiliations:** 1 Universidade Federal do Ceará, Faculdade de Medicina Programa de Pós-Graduação em Saúde Pública FortalezaCE Brasil Universidade Federal do Ceará, Faculdade de Medicina, Programa de Pós-Graduação em Saúde Pública, Fortaleza (CE), Brasil.; 2 Universidade Federal da Bahia Instituto Multidiciplinar em Saúde - Campus Anísio Teixeira Vitória da ConquistaBA Brasil Universidade Federal da Bahia, Instituto Multidiciplinar em Saúde - Campus Anísio Teixeira, Vitória da Conquista (BA), Brasil.; 3 Universidade Federal do Ceará, Centro de Ciências Departamento de Computação FortalezaCE Brasil Universidade Federal do Ceará, Centro de Ciências, Departamento de Computação, Fortaleza (CE), Brasil.; 4 Governo do Estado de Rondônia Agência Estadual de Vigilância em Saúde Porto VelhoRO Brasil Governo do Estado de Rondônia, Agência Estadual de Vigilância em Saúde, Porto Velho (RO), Brasil.; 5 Ministério da Saúde, Secretaria de Vigilância em Saúde Departamento de Doenças de Condições Crônicas e Infecções Sexualmente Transmissíveis BrasíliaDF Brasil Ministério da Saúde, Secretaria de Vigilância em Saúde, Departamento de Doenças de Condições Crônicas e Infecções Sexualmente Transmissíveis, Brasília (DF), Brasil.; 6 Universidade Federal do Ceará, Faculdade de Farmácia, Odontologia e Enfermagem Departamento de Enfermagem FortalezaCE Brasil Universidade Federal do Ceará, Faculdade de Farmácia, Odontologia e Enfermagem, Departamento de Enfermagem, Fortaleza (CE), Brasil.

**Keywords:** Hanseníase, estudos de séries temporais, análise espacial, epidemiologia, mortalidade, Brasil, Leprosy, time series studies, spatial analysis, epidemiology, Brazil, Lepra, estudios de series temporales, análisis espacial, epidemiología, Brasil

## Abstract

**Objetivo.:**

Descrever as tendências temporais e os padrões espaciais da mortalidade relacionada à hanseníase nas regiões Norte e Nordeste do Brasil de 2001 a 2017.

**Métodos.:**

Estudo ecológico misto de base populacional, de tendência temporal e espacial, baseado em dados secundários de declarações de óbito (DO) do Sistema de Informação de Mortalidade do Ministério da Saúde (SIM). As DO foram examinadas para extração dos registros de hanseníase como causa básica e associada de morte.

**Resultados.:**

Foram registrados 4 907 óbitos relacionados à hanseníase no período de interesse, 59,3% como causa associada. A hanseníase não especificada (A30.9) foi a causa mais citada nas declarações de óbito (causa básica: 72,7%; causa associada: 76,1%). Verificou-se risco acrescido de mortalidade por hanseníase em pessoas do sexo masculino, com idade ≥60 anos e de raça/cor preta ou parda. A tendência temporal por análise de pontos de inflexão (*joinpoints*) apresentou incremento na tendência geral da mortalidade na região Nordeste e nos estados de Tocantins, Maranhão, Alagoas e Bahia, assim como no sexo masculino. Para a distribuição espacial das taxas de mortalidade ajustadas por idade e sexo, assim como para as análises das médias móveis espaciais e da razão de mortalidade padronizada, padrões acima da média da área de estudo foram identificados para Acre, Rondônia, sul do estado do Pará, Tocantins, Maranhão, Piauí, sul do Ceará e regiões do norte e sul da Bahia.

**Conclusões.:**

A mortalidade por hanseníase nas regiões Norte e Nordeste é expressiva e persistente, com padrão focal de ocorrência em territórios e populações com maior vulnerabilidade. Ressalta-se a necessidade de fortalecer a atenção integral à hanseníase na rede de atenção do Sistema Único de Saúde dessas regiões.

## Abstract (en)

**Objective.:**

To describe temporal trends and spatial patterns of leprosy-related mortality in the North and Northeast of Brazil from 2001 to 2017.

**Methods.:**

This population-based, mixed ecological study employed secondary data obtained from the Health Ministry’s Mortality Information System. Death certificates were examined for extraction of information on leprosy as underlying or contributing cause of death.

**Results.:**

In the period of interest, 4 907 leprosy-related deaths were recorded. In 59.3%, leprosy was a contributing cause. “Leprosy, unspecified” (ICD-10 A30.9) was the most common cause recorded (72.7% as underlying cause; 76.1% as contributing cause). Increased risk of mortality by leprosy was observed in males, age ≥ 60 years and brown or black race/color. Joinpoint regression analysis of time trends revealed an increased overall mortality trend in the Northeast and in the states of Tocantins, Maranhão, Alagoas, and Bahia, as well as in the male sex. Regarding the spatial distribution of mortality rates adjusted by age and sex, as well as the analysis of moving spatial means and standardized mortality ratio, patterns that were above the mean for the area under study were identified for the states of Acre and Rondônia, the southern part of Pará, Tocantins, Maranhão, Piauí, south of Ceará, and north and south of Bahia.

**Conclusions.:**

Leprosy mortality in the Brazilian North and Northeast is expressive and persistent, with a focal pattern of distribution in more vulnerable territories and populations. Comprehensive leprosy care must be strengthened in the Unified Health System in these regions.

## RESUMEN

**Objetivo.:**

Describir las tendencias temporales y los patrones espaciales de la mortalidad relacionada con la lepra en las regiones norte y nordeste de Brasil del 2001 al 2017.

**Métodos.:**

Estudio ecológico mixto basado en la población, con análisis de las tendencias temporal y espacial, hecho a partir de datos secundarios tomados de las declaraciones de defunción del Sistema de Información de Mortalidad (SIM) del Ministerio de Salud. Dichas declaraciones se examinaron para extraer los registros de lepra como causa básica y asociada de defunción.

**Resultados.:**

Se registraron 4 907 defunciones relacionadas con la lepra en el período de interés, en 59,3% de las cuales se la citó como causa asociada. La “lepra no especificada” (A30.9) fue la causa más citada en las declaraciones de defunción (causa básica: 72,7%; causa asociada: 76,1%). Se verificó un mayor riesgo de mortalidad por lepra en personas de sexo masculino, mayores de 60 años y de raza o de piel negra o morena. La tendencia temporal por análisis de puntos de inflexión (joinpoints) mostró un incremento en la tendencia general de la mortalidad en la región nordeste y en los estados de Tocantins, Maranhão, Alagoas y Bahía, así como en personas de sexo masculino. En lo referente a la distribución espacial de las tasas de mortalidad ajustadas por edad y sexo, así como a los análisis de las medias móviles espaciales y de la razón de mortalidad normalizada, se identificaron patrones superiores a la media de la zona de estudio en Acre, Rondônia, el Sur del estado de Pará, Tocantins, Maranhão, Piauí, el Sur de Ceará y las regiones del Norte y Sur de Bahía.

**Conclusiones.:**

La mortalidad por lepra en las regiones norte y nordeste es significativa y persistente, con un patrón focal de incidencia en los territorios y poblaciones con mayor vulnerabilidad. Se hace hincapié en la necesidad de fortalecer la atención integral a esta enfermedad en la red de atención del Sistema Único de Salud de esas regiones.

Entre as doenças tropicais negligenciadas (DTN), a hanseníase é a que apresenta maior risco de gerar incapacidade física (1); aproximadamente 5% das pessoas acometidas são diagnosticadas com incapacidade física de grau 2 (GIF2) (2). Como doença crônica em áreas endêmicas com alta vulnerabilidade, a hanseníase está associada a diagnóstico tardio, o que contribui para síndromes clínicas mais complexas, entre elas as associadas a infecções e lesões osteomusculares, além de eventos imunológicos, muitas vezes graves, com impacto negativo na qualidade de vida (3, 4). Por sua vez, a associação entre infecção por *Mycobaterium leprae* e coinfecções – por exemplo, vírus da imunodeficiência humana (HIV), vírus linfotrópico de células T humanas tipo 1 (HTLV-1) e vírus da hepatite B (HBV) e hepatite C (HCV) –, em vigência ou não de alterações imunológicas, podem levar a evolução mais grave da hanseníase, inclusive óbito (5, 6).

Entretanto, o óbito por hanseníase tem sido negligenciado como objeto de pesquisa e de ações de vigilância e controle, impedindo que importantes lacunas no conhecimento sejam preenchidas (7, 8). Estudos sobre a mortalidade relacionada à doença indicam uma associação do óbito com complicações relacionadas principalmente ao manejo de episódios reacionais, especialmente com corticosteroides (7, 8). O uso de corticosteroides em doses imunossupressoras e por longos períodos tem sido associado a complicações sistêmicas graves, como sepse, glomerulonefrite proliferativa aguda fatal, síndrome de hiperinfecção por *Strongyloides stercoralis*, broncopneumonia, tuberculose e insuficiência cardíaca ou renal, em particular nefrite intersticial (6-9). A poliquimioterapia (dapsona, rifampicina e clofazimina), comum no tratamento da hanseníase, também tem sido associada a óbito, agranulocitose fatal (dapsona), síndrome de hipersensibilidade à dapsona (SHD) possivelmente induzida por rifampicina, além de hepatite, nefrite túbulo-intersticial, miocardite e efeitos tóxicos intestinais, como enteropatia fatal (6, 7).

A análise de DTN como causa básica de mortalidade (76 847 óbitos) no país de 2000 a 2011 evidenciou 3 156 (4,1%) óbitos por hanseníase, quinta principal causa, com taxa de mortalidade ajustada por idade semelhante à observada para dengue e leishmanioses (0,16 [0,15–0,18]/100 000 habitantes)(10). A junção de causas associadas de morte às causas básicas ampliou em 23 967 (23,8%) o número de óbitos por DTN, sendo a hanseníase a terceira principal DTN (7,6%; 7 732 óbitos) (11).

Por apresentar o segundo maior número de casos novos de hanseníase no contexto mundial (12), o Brasil tem significativa importância no cenário global de manejo dessa doença. De 2012 a 2016, o país registrou 151 764 casos, com média anual de 2 042 casos novos com deformidades visíveis (GIF2) no diagnóstico (13). Mesmo com a redução de quase 40 000 casos em 2008 para aproximadamente 26 000 casos em 2017 (>30%), persistem os desafios para o controle da doença (14) e para o pós-alta da poliquimioterapia (12). Historicamente, as regiões Norte, Nordeste e Centro-Oeste mantêm-se com número elevado de casos novos de hanseníase, além de apresentarem as maiores taxas de detecção geral e de GIF2 (13). Tocantins, Rondônia, Pará e Maranhão são os estados com as maiores taxas de detecção geral, detecção em menores de 15 anos e de GIF2 (13, 15).

Dado esse contexto, o presente trabalho objetiva descrever de forma integrada as tendências temporais e os padrões espaciais da mortalidade relacionados à hanseníase nas regiões Norte e Nordeste do Brasil, de 2001 a 2017.

## MATERIAIS E MÉTODOS

**FIGURA 1.**
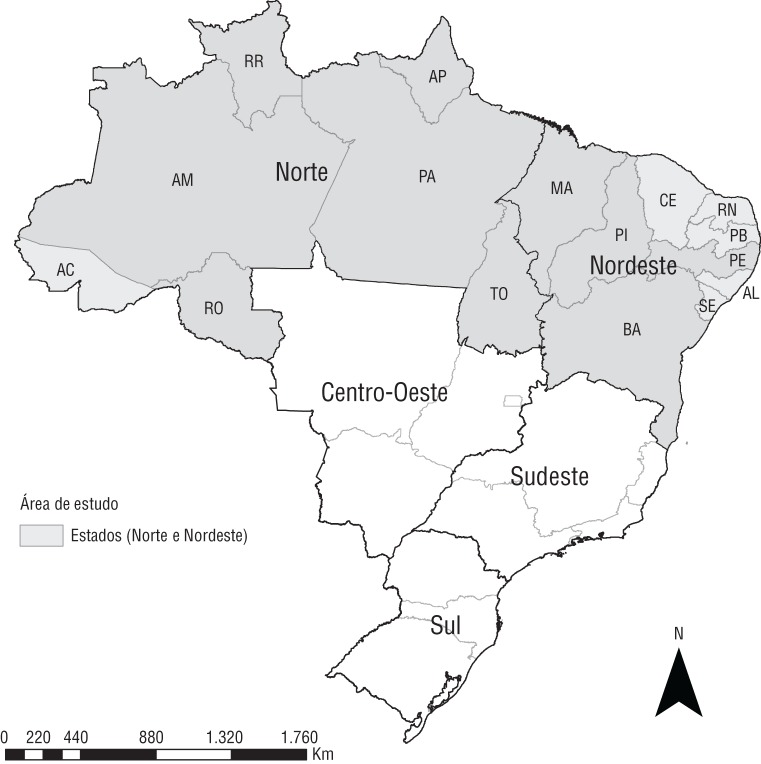
Áreas de estudo: estados e regiões Norte e Nordeste do Brasil

O presente estudo ecológico de base populacional com desenho misto, de tendência temporal e espacial, baseou-se em dados secundários registrados nas declarações de óbito (DO) em que a hanseníase foi mencionada como causa básica ou associada de morte (causas múltiplas de morte). Foram analisadas as DO de 2001 a 2017 nas regiões Norte e Nordeste do Brasil (16) (figura 1). Essas regiões englobam 16 estados e 2 244 municípios, com área total de 5 408 131 km^2^.

O Nordeste apresenta densidade demográfica de 34,15 habitantes por km^2^ – 8 vezes a densidade da região Norte, com 4,12 habitantes por km^2^. No censo de 2010, a população da região Norte era de 15 864 454 habitantes, com projeção de 18 182 253 habitantes para 2018. O índice de Gini, que mede o grau de concentração de renda (variando de 0 - completa igualdade – a 1 - completa desigualdade), teve evolução positiva, tendo caído de 0,62 em 2010 para 0,50 em 2015; o índice de vulnerabilidade social (IVS) caiu de 0,438 (alto) em 2010 para 0,298 (baixo) em 2015 (17, 18). No Nordeste, a população em 2010 era de 53 081 950 habitantes, com projeção de 56 760 780 para 2018 (3 vezes maior que a do Norte). De 2010 para 2015, o índice de Gini caiu de 0,62 para 0,51, e o IVS, de 0,408 (alto) para 0,311 (médio) (17-19).

As causas de óbito foram identificadas e registradas utilizando os códigos da 10ª revisão da Classificação Estatística Internacional de Doenças e Problemas Relacionados à Saúde (CID-10) da Organização Mundial da Saúde (OMS) (20). As causas de morte relacionadas à hanseníase analisadas foram: A30, hanseníase; A30.0, hanseníase indeterminada; A30.1, hanseníase tuberculoide; A30.2, hanseníase tuberculoide borderline; A30.3, hanseníase dimorfa; A30.4, hanseníase lepromatosa borderline; A30.5, hanseníase lepromatosa; A30.8, outras formas de hanseníase; A30.9, hanseníase não especificada; e B92, sequelas de hanseníase (17).

Com base nos registros das DO e nas definições vigentes, foram identificadas as “causas básicas” de óbito com menção à hanseníase. A causa básica é definida como o evento único, doença ou lesão que iniciou a sequência de acontecimentos que levaram diretamente à morte, ou ainda como circunstância do acidente ou das violências que produziram a lesão fatal. As “causas associadas”, diferentes da causa básica, são fatores que contribuíram significativamente, direta ou indiretamente, para a morte (7, 19).

Os dados de 2001 a 2017 do Sistema de Informação de Mortalidade do Ministério da Saúde (SIM-MS), no Departamento de Informática do SUS (DATASUS), foram obtidos via protocolo formal junto à Coordenação Geral de Informações e Análises Epidemiológicas (CGIAE) do Departamento de Vigilância de Doenças e Agravos não Transmissíveis e Promoção da Saúde da Secretaria de Vigilância em Saúde do Ministério da Saúde (DANTPS/SVS/MS) (http://www2.datasus.gov.br/DATASUS/index.php?area=0901&item=1&acao=26&pad=31655). A despeito de serem dados públicos, os dados de 2017 ainda não estavam disponíveis no momento das análises, o que demandou a solicitação da base de dados sem variáveis de identificação das pessoas via protocolo junto ao Ministério da Saúde. Os dados populacionais, obtidos no DATASUS (http://tabnet.datasus.gov.br/cgi/deftohtm.exe?ibge/cnv/popuf.def), têm como fonte os censos nacionais de 2000 e 2010 e as estimativas populacionais de anos intercensitários (2001 a 2009 e 2011 a 2015) fornecidas pelo Instituto Brasileiro de Geografia e Estatística (IBGE). O método de interpolação de dados foi utilizado para 2016 e 2017.

***Fonte:*** Bases cartográficas (shapefiles) IBGE 2013, com mapas.

Região Norte: Amazonas (AM), Roraima (RR), Amapá (AP), Pará (PA), Tocantins (TO), Rondônia (RO), Acre (AC). Região Nordeste: Maranhão (MA), Piauí (PI), Ceará (CE), Rio Grande do Norte (RN), Paraíba (PB), Pernambuco (PE), Alagoas (AL), Sergipe (SE).

O IVS foi obtido a partir de dados do Atlas de Desenvolvimento do Brasil e do Instituto de Pesquisa Econômica Aplicada (IPEA). Para o cálculo do IVS, foram integrados 16 indicadores da Plataforma do Atlas de Desenvolvimento Humano, que está organizado em três dimensões: infraestrutura urbana, capital humano e renda e trabalho (18).

Realizou-se análise por frequência simples e relativa, com cálculo do risco relativo (RR) e dos intervalos de confiança de 95% (IC95%) e determinação de diferenças entre grupos pelo teste do qui-quadrado (χ^2^) de Pearson. Para o cálculo das taxas médias brutas e ajustadas por idade e sexo (com IC95%), foi utilizado o método direto de padronização, com taxas estratificadas por região (Norte e Nordeste) e estado de residência, sexo/gênero (feminino, masculino), faixa etária em anos (0–14, 15–59, ≥60), raça/cor (branca, preta, amarela, parda, indígena), porte da cidade (pequeno porte I: ≤ 20 000 habitantes, pequeno porte II: de 20 001 a 50 000 habitantes, médio porte: 50 001 a 100 000 habitantes, grande porte: > 100 001 habitantes) (21, 22) e IVS (muito baixo: 0,00 a 0,199; baixo, 0,200 a 299; médio, 0,300 a 0,399; alto: 0,400 a 0,499; muito alto: 0,500 a 1,00). As taxas tiveram como base a população do censo de 2010 e foram expressas por 100 000 habitantes.

Para o cálculo das tendências temporais, o *software* Joinpoint Regression versão 4.4.2 foi utilizado. Realizou-se regressão de Poisson por pontos de inflexão (*joinpoints*), com significância estatística determinada pelo método de permutação de Monte Carlo para reconhecimento da melhor linha de cada seguimento. Posteriormente, foram testadas e validadas a variação percentual anual (*annual percent change*, APC) e a variação percentual anual média (*average annual percent change*, AAPC) com IC95%. Finalmente, verificou-se a ocorrência de padrões de crescimento (APC positivas), de redução (APC negativas) ou de ausência de tendência (APC igual a zero) (22).

Para a análise da distribuição espacial, as taxas médias dos períodos de 2001 a 2005, 2006 a 2010 e 2011 a 2017 (2, 8) foram calculadas e padronizadas pelo método direto, segundo a estrutura etária por sexo do censo de 2010 (por 100 000 habitantes). Para a identificação de padrões de concentração de óbitos, foram calculadas taxas pela média móvel espacial, considerando os óbitos de municípios vizinhos. Buscou-se identificar municípios com número de óbitos acima do esperado (excesso de risco) pela razão de mortalidade padronizada e procedeu-se à divisão de óbitos registrados por óbitos esperados, uma técnica de abordagem não espacial que ignora o efeito da autocorreção espacial.

Para a categorização das classes espaciais das taxas ajustadas e da média móvel espacial, utilizou-se o método de quebras naturais do algoritmo de classificação de Jenks (*natural breaks*). O município de residência do óbito foi utilizado como unidade de análise (n = 2 244 municípios, conforme divisão territorial de 2013). Foram excluídos óbitos com município de residência desconhecido.

As análises estatísticas foram realizadas com o *software* Stata versão 11.2. Para a análise espacial, o cálculo de indicadores de autocorrelação e a construção de mapas temáticos foram utilizados o qGis versão 2.18.6 e o GeoDa versão 1.8.16.4.1.

O presente estudo foi baseado em dados secundários de mortalidade de natureza anônima, disponíveis publicamente e obtidos formalmente junto ao Ministério da Saúde, sendo dispensada a aprovação ética.

## RESULTADOS

Nos 17 anos da análise, foram registrados 4 907 óbitos com menção de hanseníase em qualquer campo da DO nas regiões Norte e Nordeste do país – 1 999 (40,7%) como causa básica e 2 908 (59,3%) como causa associada. A “hanseníase não especificada” (A30.9) foi a causa de óbito mais frequente, tanto como causa básica (n = 1 453, 72,7%) quanto como causa associada (n = 3 764, 76,1%), seguida por “sequelas de hanseníase” (B92) (n = 268, 13,4% e n = 602, 12,2%, respectivamente) (tabela 1).

A taxa de mortalidade ajustada por idade e sexo foi de 0,41 (0,36 a 0,45) por 100 000 habitantes. A região Nordeste teve maior número de óbitos (n = 3 572, 72,8%). Entretanto, a taxa de mortalidade da região Norte foi quase duas vezes maior: 0,60 (0,46 a 0,73) por 100 000 habitantes (tabela 2).

No Norte, o Pará registrou o maior número de óbitos (n = 573, 42,9%), enquanto o Tocantins registrou a maior taxa de mortalidade ajustada (1,22 [0,61 a 1,82] por 100 000 habitantes). No Nordeste, o Maranhão teve o maior número de óbitos (n = 947; 26,5%) e a maior taxa de mortalidade (0,92 [0,68 a 1,16]/100 000 habitantes).

**TABELA 1.** Hanseníase como causa de óbito básica ou associada nas regiões Norte e Nordeste, Brasil, 2001 a 2017

Código CID-10	n (%)		
	Causa básica	Causa associada	Causa múltipla (básica + associada)
Hanseníase indeterminada (A30.0)	62 (3,1)	148 (3,0)	210 (3,0)
Hanseníase tuberculoide (A30.1)	26 (1,3)	37 (0,7)	63 (0,9)
Hanseníase tuberculoide borderline (A30.2)	4 (0,2)	10 (0,2)	14 (0,2)
Hanseníase dimorfa (A30.3)	35 (1,8)	73 (1,5)	108 (1,6)
Hanseníase lepromatosa borderline (A30.4)	6 (0,3)	15 (0,3)	21 (0,3)
Hanseníase lepromatosa (A30.5)	104 (5,2)	220 (4,4)	324 (4,7)
Outras formas de hanseníase (A30.8)	41 (2,1)	75 (1,5)	116 (1,7)
Hanseníase não especificada (A30.9)	1 453 (72,7)	3 764 (76,1)	5 217 (75,1)
Sequelas de hanseníase (B92)	268 (13,4)	602 (12,2)	870 (12,5)
Total	1 999 (100,0)^a^	4 944 (100,0)^a^	6 943 (100,0)^a^

^a^ Declarações de óbito que registraram pelo menos uma causa de morte relacionada à hanseníase.

Os óbitos no sexo masculino representaram 73,4% (n = 3 604) do total, com taxa de mortalidade ajustada de 0,65 (0,57 a 0,74) por 100 000 habitantes e RR de 2,88 (IC95%: 2,22 a 3,75; *P*-valor < 0,0001). O número de óbitos foi maior no grupo com mais de 60 anos (n = 2 650; 54%), com taxa de mortalidade ajustada de 2,78 (3,34 a 4,98) por 100 000 habitantes e RR de 7,90 (IC95%: 6,26 a 9,96; *P*-valor < 0,0001). Pessoas da raça/cor parda representaram 62,4% (n = 3 061) da população, com taxa de mortalidade ajustada de 0,45 (0,38 a 0,51) por 100 000 habitantes. Maior RR foi verificado na raça/cor preta, mas sem significância (1,17; IC95%: 0,79 a 1,72; *P*-valor = 0,4302) (tabela 2).

Municípios de grande porte apresentaram maior número de óbitos (n = 2 359; 48,1%), taxa de mortalidade ajustada de 0,47 (0,41 a 0,57) por 100 000 habitantes e RR de 1,09 (IC95%: 0,78 a 1,53; *P*-valor = 0,5973), porém sem significância estatística. O conjunto de municípios na categoria de IVS médio (n = 2 025; 41,3%) teve maior número de óbitos, mas a taxa de mortalidade ajustada foi maior entre aqueles com IVS muito baixo (0,49 [0,32–0,67] por 100 000 habitantes) (tabela 2).

Na análise integrada das regiões, o período de 2001 a 2009 apresentou tendência temporal de aumento da mortalidade (APC 4,3 [IC95%: 1,0 a 7,7]). O Nordeste apresentou tendência de aumento de 2001 a 2006 (APC 8,6 [IC95%: 1,8 a 15,9]), enquanto o Norte não apresentou tendência definida (APC 0,2 [IC95%: -1,7 a 2,2]) (tabela 3). Os estados de Tocantins (2001 a 2017: APC 6,1 [IC95%: 2,0 a 10,4]), Maranhão (2001 a 2007: APC 14,8 [IC95%: 5,4 a 25,0]), Alagoas (2001 a 2017, APC 4,0 [IC95%: 0,3 a 7,9]) e Bahia (APC 3,6 [IC95%: 0,8 a 6,5]) apresentaram tendência de aumento; os demais estados não apresentaram tendência definida.

De 2001 a 2009, verificou-se tendência de aumento da mortalidade no sexo masculino (APC 5,1 [IC95%: 1,5 a 8,8]), enquanto o sexo feminino não apresentou tendência definida (APC 0,0 [IC95%: -1,5 a 1,4]). Pessoas com idade ≥ 60 anos apresentaram tendência de aumento de 2001 a 2009 (APC 4,7 [IC95%: 0,6 a 9,0]), mas redução de 2009 a 2017 (APC -4,0 [IC95%; -7,4 a -0,5]). A raça/cor parda apresentou tendência de aumento de 2001 a 2009 (APC 6,4 [IC95%: 3,2 a 9,6]); as demais categorias de raça/cor não apresentaram tendência definida (tabela 3).

No período geral, verificou-se tendência de aumento da mortalidade em municípios de pequeno (APC 4,8 [IC95%: 2,6 a 7,1]) e médio porte (APC 2,9 [IC95%: 1,5 a 4,4]) e tendência de redução em municípios de grande porte (APC -1,4 [IC95%: -2,5 a -0,3]). Municípios com IVS “muito alto” apresentaram tendência de aumento de 2001 a 2008 (APC 15,7 [IC95%: 6,5 a 25,8]), com as demais faixas de IVS sem tendência definida (tabela 3).

**TABELA 2.** Taxa de mortalidade relacionada à hanseníase ajustada por idade/sexo por 100 000 habitantes, Norte e Nordeste, Brasil, 2001 a 2017

Variável^a^	No. (%)	Taxa ajustada (IC95%)	RR^b^ (IC95%)	*P*-valor^c^
Total	4 907 (100)	0,41 (0,36 a 0,45)		
Região				
Norte	1 335 (27,2)	0,60 (0,46 a 0,73)	1	
Nordeste	3 572 (72,8)	0,37 (0,32 a 0,42)	0,79 (0,61 a 1,03)	0,0813
Sexo				
Feminino	1 300 (26,5)	0,20 (0,15 0,24)	1	
Masculino	3 604 (73,4)	0,65 (0,57 a 0,74)	2,88 (2,22 a 3,75)	<0,0001
Faixa etária (anos)								
0 a 14	28 (0,6)	0,01 (0,00 a 0,02)	0,03 (0,01 a 0,14)	<0,0001
15 a 59	2 222 (45,3)	0,30 (0,24 a 0,35)	1	
≥ 60	2 650 (54,0)	2,78 (2,34 a 3,22)	7,90 (6,26 a 9,96)	<0,0001
Raça/cor								
Branca	929 (18,9)	0,25 (0,18 a 0,32)	0,68 (0,50 a 0,91)	0,0109
Preta	508 (10,4)	0,40 (0,26 a 0,55)	1,17 (0,79 a 1,72)	0,4302
Amarela	17 (0,3)	0,00 (0,00 a 0,00)	0,29 (0,04 a 2,08)	0,2188
Parda	3 061 (62,4)	0,45 (0,38 a 0,51)	1	
Indígena	10 (0,2)	0,00 (0,00 a 0,00)	0,46 (0,06 a 3,27)	0,4357
Porte municipal				
Pequeno porte I	793 (16,2)	0,31 (0,22 a 0,40)	0,81 (0,54 a 1,22)	0,3054
Pequeno porte II	994 (20,3)	0,34 (0,25 a 0,43)	0,84 (0,57 a 1,25)	0,3943
Médio porte	759 (15,5)	0,43 (0,31 a 0,56)	1	
Grande porte	2.359 (48,1)	0,49 (0,41 a 0,57)	1,09 (0,78 a 1,53)	0,5973
IVS				
Baixo	562 (11,5)	0,49 (0,32 a 0,67)	1,12 (0,76 a 1,64)	0,5709
Médio	2 025 (41,3)	0,44 (0,36 a 0,52)	1	
Alto	1 360 (27,7)	0,36 (0,28 a 0,44)	0,86 (0,65 a 1,14)	0,3017
Muito alto	958 (19,5)	0,39 (0,28 a 0,49)	0,83 (0,60 a 1,14)	0,2425

^a^ Dados indisponíveis: sexo: 3; idade: 7; raça/cor: 382; porte municipal: 2; IVS: 2. Porte municipal: pequeno porte I, ≤ 20 000 habitantes; pequeno porte II, de 20 001 a 50 000 habitantes; médio porte, 50 001 a 100 000 habitantes; grande porte: >100 001 habitantes. IVS = índice de vulnerabilidade social; muito baixo: 0,00 a 0,199; baixo, 0,200 a 299; médio, 0,300 a 0,399; alto: 0,400 a 0,499; muito alto: 0,500 a 1,00.

^b^ Risco relativo.

^c^ *P*-valor: teste χ^2^ de Pearson.

**FIGURA 2.**
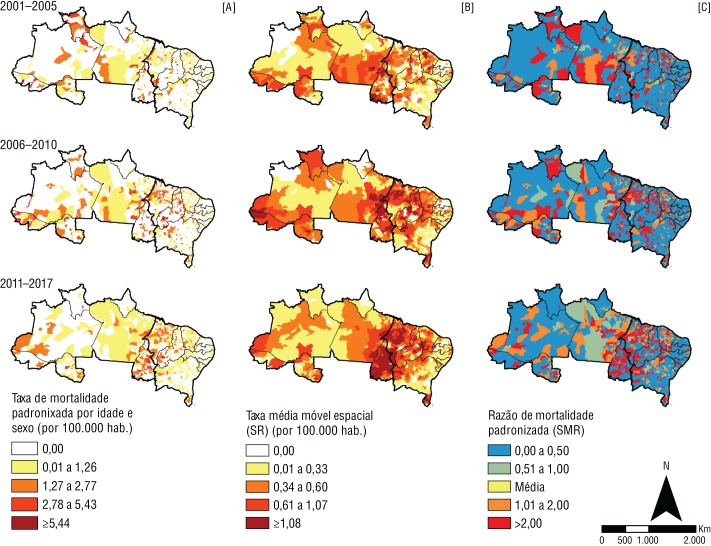
Distribuição da mortalidade relacionada à hanseníase nas regiões Norte e Nordeste do Brasil, 2001 a 2017

^a^ A) Mapas da taxa ajustada por idade e sexo/100 000 habitantes; B) mapas das taxas de médias móveis espaciais (SR) /100 000 habitantes; C) mapas das razões de mortalidade padronizadas (SMR).

Em munícipios de Roraima e no norte e sul do estado do Amazonas, verificou-se concentração das taxas de mortalidade ajustadas por idade e sexo e redução de municípios com taxas elevadas de mortalidade (figura 2A). Tocantins, Maranhão, Piauí, Bahia e o sul do Pará apresentaram aumento do número de municípios com altas taxas; os demais estados não apresentaram tendências definidas de concentração de municípios e taxas (figura 2A).

Nos primeiros 10 anos da análise, verificou-se concentração de altas taxas de mortalidade calculadas pela média móvel espacial nos estados de Roraima, Tocantins, Maranhão, Piauí, Ceará, nas regiões norte e sul do Amazonas, no sul do Pará e no norte e sul da Bahia. Em Rondônia, taxas elevadas de mortalidade foram registradas durante todo o período (figura 2B).

Houve ampliação na concentração de municípios com razões de mortalidade padronizada acima da média em áreas específicas de Roraima, do sul do Pará e do Amazonas, do norte de Rondônia, do centro-norte do Maranhão, do sul do Ceará, do norte e sul da Bahia. No estado de Tocantins, razões acima da média vêm sendo observadas ao longo do tempo, ocupando o centro do estado no período de 2011 a 2017 (figura 2C).

## DISCUSSÃO

A mortalidade relacionada à hanseníase persiste como problema de saúde pública no Norte e Nordeste do Brasil, regiões de alta endemicidade no Brasil. A presente análise integrada detectou taxas de mortalidade com tendência geral de aumento mais expressivo no Nordeste e maior risco para homens, idosos e pessoas de raça/cor preta. Análises espaciais e espaço-temporais evidenciaram altas taxas de mortalidade em áreas relacionadas à elevada vulnerabilidade social (11, 23, 24).

Uma questão que se apresenta é a diferença entre os padrões de cobertura e a qualidade da vigilância do óbito no país, em particular nas regiões analisadas no presente estudo (7, 8, 11, 25, 26). As causas mal definidas tiveram elevada proporção no período da análise, principalmente na causa básica. No Brasil, um estudo anterior mostrou que, para óbitos que tiveram a hanseníase como causa múltipla, o percentual de causas mal definidas foi semelhante (7). Isso reforça a hipótese de falhas potenciais na investigação do óbito, com baixa cobertura e qualidade (19). Entretanto, mesmo com potencial de subdimensionamento, a análise da hanseníase como causa de óbito é relevante, tanto pela endemicidade quanto para preencher as lacunas de conhecimento existentes (7-9).

**TABELA 3.** Tendência temporal conforme análise de pontos de inflexão da taxa bruta de mortalidade relacionada à hanseníase, Norte e Nordeste, Brasil, 2001 a 2017

Variável	Tendência	
	Período	APC (IC95%)
Total	2001 a 2009	4,3^a^ (1,0 a 7,7)
	2009 a 2017	-1,5 (-4,3 a 1,4)
Região		
Norte	2001 a 2017	0,2 (-1,7 a 2,2)
Nordeste	2001 a 2006	8,6^a^ (1,8 a 15,9)
	2006 a 2017	-0,4 (-2,1 a 1,3)
Sexo		
Feminino	2001 a 2017	0,0 (-1,5 a 1,4)
Masculino	2001 a 2009	5,1^a^ (1,5 a 8,8)
	2009 a 2017	-1,2 (-4,1 a 1,8)
Faixa etária (anos)		
0 a 14	2001 a 2017	-6,3 (-12,4 a 0,3)
15 a 59	2001 a 2017	-0,2 (-1,5 a 1,2)
≥60	2001 a 2009	4.7^a^ (0,6 a 9,0)
	2009 a 2017	-4.0^a^ (-7,4 a -0,5)
Raça cor		
Branca	2001 a 2017	0,7 (-1,0 a 2,4)
Preta	2001 a 2017	-1,3 (-4,1 a 1,6)
Amarela	2001 a 2017	-15,4^a^ (-21,6 a -8,8)
Parda	2001 a 2009	6,4^a^ (3,2 a 9,6)
	2009 a 2017	-0,8 (-3,2 a 1,7)
Indígena	2001 a 2003	-53,8 (-86,3 a 56,2)
	2003 a 2017	5,7 (-1,4 a 13,3)
Porte municipal^b^		
Pequeno porte I	2001 a 2017	4,8^a^ (2,6 a 7,1)
Pequeno porte II	2001 a 2007	14,4^a^ (4,3 a 25,4)
	2007 a 2017	-0,7 (-4,0 a 2,8)
Médio porte	2001 a 2017	2,9^a^ (1,5 a 4,4)
Grande porte	2001 a 2017	-1,4^a^ (-2,5 a -0,3)
IVS^c^		
Baixo	2001 a 2017	0,6 (-1,3 a 2,6)
Médio	2001 a 2017	-0,9 (-2,1 a 0,4)
Alto	2001 a 2017	2,0 (-0,3 a 4,3)
Muito alto	2001 a 2008	15,7^a^ (6,5 a 25,8)
	2008 a 2017	0,2 (-3,9 a 4,5)

^a^ Significativamente diferente de 0 (*P* < 0,05), método de permutação de Monte Carlo.

^b^ Pequeno porte I, ≤ 20 000 habitantes; pequeno porte II, de 20 001 a 50 000 habitantes; médio porte, 50 001 a 100 000 habitantes; grande porte: >100 001 habitantes.

^c^ IVS = índice de vulnerabilidade social; muito baixo: 0,00 a 0,199; baixo, 0,200 a 299; médio, 0,300 a 0,399; alto: 0,400 a 0,499; muito alto: 0,500 a 1,00.

Em 2005, foi implementado no Brasil um projeto nacional a fim de reduzir registros de óbitos por causas mal definidas. Em 2000, a proporção de causas mal definidas era de 14,3% (25), passando para 7,0% em 2010, sendo as maiores proporções nas regiões Norte e Nordeste (11,8% e 7,8%, respectivamente) (26). Entretanto, mesmo com essa melhoria, problemas como imprecisão nos registros da DO por médicos e ausência de serviços de verificação de óbito, entre outros, podem fragilizar a investigação dos óbitos (7, 8).

Neste estudo, as causas mal definidas foram seguidas por “sequelas da hanseníase”, também indicando falhas operacionais no diagnóstico, tratamento e seguimento (7-9). Estudos realizados no Brasil e em estados endêmicos indicam percentuais de óbitos por sequelas de 5 a 11%, mais evidentes nas causas associadas (7, 8). Esse aspecto pode indicar falhas no alcance da atenção integral do Sistema Único de Saúde (SUS), especialmente após a alta da poliquimioterapia (12, 27), devido às restrições de acesso à rede de atenção básica e especializada. Isso pode gerar progressão de lesões associadas à hanseníase, eventualmente levando ao óbito (4, 6-8, 27). Um exemplo é o reconhecimento de sepse como principal causa de óbito associada à hanseníase de 1999 a 2007 (6, 7). O contexto de diagnóstico tardio de casos novos da doença verificado nessas regiões amplia o risco de desenvolvimento de síndromes clínicas ainda mais graves (2-4, 6).

A proporção de óbitos na região Nordeste foi superior à do Norte, possível reflexo do número de casos novos notificados. As taxas de mortalidade foram superiores no Norte, mesmo com taxas semelhantes de detecção de casos novos (13), pela diferença da população sob risco. Para o Brasil como um todo, a proporção de óbitos de 2000 a 2011 foi semelhante nas duas regiões, com risco acrescido quando comparadas à região Sudeste (7). Pará, Maranhão, Ceará, Pernambuco e Bahia apresentaram maior número de óbitos, com taxas mais elevadas no Acre, Tocantins, Maranhão e Piauí. Taxas de mortalidade elevadas também foram observadas nos mesmos estados em um estudo que abordou a mortalidade por hanseníase no país de 2000 a 2011 (7) na Bahia (8).

O aumento da tendência de mortalidade em homens reafirma a relevância do sexo como caraterística a ser considerada no processo de adoecimento (28). Tais padrões também são observados para DTN em geral no Brasil (10), mas sem tendência significativa para hanseníase (7). Entretanto, um estudo sobre a mortalidade da hanseníase na Bahia mostrou tendência de aumento para homens (8), população em que mais ocorrem situações de abandono de tratamento e de recidivas (29).

Considerando o caráter crônico da hanseníase, o maior risco da mortalidade na população de maior idade indica baixa qualidade de vida e maior impacto de comorbidades, infecciosas ou não (5, 6, 8). As condições físicas e imunológicas dessa faixa etária ampliam consideravelmente a vulnerabilidade para desfechos desfavoráveis (7, 12, 14, 30). As pessoas idosas com alta proporção de comorbidades são as mais afetadas, em particular homens (39,18 casos novos por 100 000 habitantes vs. 21,21 casos novos por 100 000 habitantes para mulheres) (13).

A abordagem integrada de DTN pode potencializar ações de controle, como mostra a experiência brasileira com hanseníase, tracoma e esquistossomose (23). O reconhecimento da superposição de casos e óbitos por DTN em um mesmo município tem sido uma estratégia importante com vistas a novas estratégias de integração de ações de controle. Em 2015, estados com taxas elevadas de mortalidade por DTN tiveram altas taxas de hanseníase, superadas apenas pela doença de Chagas nos estados de Goiás, Distrito Federal e Minas Gerais (23).

No Brasil, taxas mais elevadas de detecção geral com GIF2 foram registradas em pessoas com 60 anos ou mais e em casos multibacilares, principalmente em homens (13, 29). Nessa mesma população, verificou-se alto percentual de casos com reações hansênicas em cidades endêmicas do Nordeste, com maior frequência em formas multibacilares com uso de poliquimioterapia (27). Para as DTN, ser idoso é um fator de risco para morte (10), como apontado na avaliação da esperança de vida corrigida pela incapacidade (DALY): no Brasil, em 2016, taxas mais altas concentravam-se em faixas etárias mais avançadas (24). Dada a imunossenescência, instituir o cuidado longitudinal incluindo essa idade é uma ação prioritária.

O maior percentual de óbitos no presente estudo foi observado em pessoas da raça/cor parda, possivelmente refletindo a desigualdade social (29). Outro estudo em um estado endêmico do Nordeste do Brasil evidenciou aumento das taxas de mortalidade por hanseníase na população parda (8). Além dos determinantes sociais, outros fatores contribuem para o aumento da gravidade clínica e mortalidade nessa população, como padrões diferenciais de moduladores da resposta imune (30), a exemplo da vitamina D (31).

A tendência de aumento em municípios com menos de 100 000 habitantes pode ser explicada não apenas pelos níveis de pobreza, como verificado no Norte do Brasil (2), mas também por questões operacionais da rede de atenção à saúde (2, 8, 29, 32). Estudo realizado no Sudeste do Brasil identificou associação entre altas taxas de detecção e IVS altos/muito altos (33). Por outro lado, mortalidade mais expressiva foi verificada em municípios com IVS médio, corroborando achados no município de Juiz de Fora, Sudeste do Brasil, onde taxas mais altas estavam em IVS baixos (33). Entretanto, para valores de IVS muito altos, a tendência verificada foi de aumento. No Brasil, em 2015, verificou-se relação diretamente proporcional entre taxas de detecção de casos e óbitos DTN com IVS (23).

Taxas de mortalidade ajustadas, taxas de médias móveis espaciais e razões de mortalidade padronizadas apresentaram padrões de distribuição semelhantes aos relatados em outros estudos no país (7, 23). As evidências de áreas com altas taxas de mortalidade por DTN corroboram os achados do presente estudo, excetuando-se os casos de Minas Gerais e Goiás, pelo efeito da doença de Chagas (7). A atuação em municípios com maior risco e vulnerabilidade de morte por hanseníase poderá ser usada como estratégia para ampliar o impacto das ações de controle de DTN.

O presente estudo apresenta limitações relativas aos métodos, considerando que o uso de dados secundários pode levar à subestimação pela incompletude e inconsistências nas bases de origem. Além disso, o padrão diferencial de vigilância de óbitos no país coloca as regiões Norte e Nordeste em contextos de menor cobertura e qualidade na investigação de óbitos. Entretanto, a análise de uma série histórica de 17 anos, com representatividade macrorregional em áreas de alta endemicidade, diante de limitadas evidências científicas populacionais, justifica a realização do estudo.

Em síntese, a mortalidade por hanseníase nas regiões Norte e Nordeste do Brasil persiste como grave problema de saúde pública. O reconhecimento de áreas com tendência de aumento ou não, definida em territórios e populações específicas (homens, idosos, raça/cor parda ou preta), reforça a necessidade de fortalecer ações de atenção e vigilância. Por ser ligada a baixas condições socioeconômicas e pobreza, a mortalidade por hanseníase é uma questão crítica em municípios com vulnerabilidade social. Assim, justifica-se o desenvolvimento de políticas intersetoriais para superação das graves desigualdades sociais.

### Financiamento.

AFF foi bolsista de mestrado do Conselho Nacional de Desenvolvimento Científico e Tecnológico (CNPq). GSMG é bolsista de doutorado da Fundação Cearense de Apoio ao Desenvolvimento Científico e Tecnológico do Estado do Ceará (FUNCAP-CE). ASR foi bolsista de mestrado da Coordenação de Aperfeiçoamento de Pessoal de Nível Superior (CAPES).

### Contribuição dos autores.

AFF, EAS e ANR contribuíram na concepção do projeto, análise e interpretação dos dados, redação do artigo, revisão crítica e aprovação da versão a ser publicada. MSL, GSM, FC, ESNA, SASN, CRF, ASR e LGT colaboraram na análise e interpretação dos dados, redação do artigo, revisão crítica e aprovação final da versão a ser publicada.

### Declaração.

As opiniões expressas no manuscrito são de responsabilidade exclusiva dos autores e não refletem necessariamente a opinião ou política da RPSP/PAJPH ou da Organização Pan-Americana da Saúde (OPAS).
